# 537. Safety and Effectiveness of Ensitrelvir for the Treatment of COVID-19 in Japanese Clinical Practice: A Post-marketing Surveillance (Interim Analysis)

**DOI:** 10.1093/ofid/ofad500.606

**Published:** 2023-11-27

**Authors:** Eriko Ogura, Noriko Nakagawa, Noriko Hayashi, Eri Tsukimura, Satoru Takashima

**Affiliations:** Shionogi & Co., Ltd., Osaka, Osaka, Japan; Shionogi, Osaka, Osaka, Japan; Shionogi & Co., Ltd., Osaka, Osaka, Japan; Shionogi Pharmacovigilance Center Co., Ltd., Osaka, Osaka, Japan; Shionogi & Co., Ltd., Osaka, Osaka, Japan

## Abstract

**Background:**

Ensitrelvir fumaric acid (ensitrelvir) is a novel anti-SARS-CoV-2 drug and an inhibitor of SARS-CoV-2 3CL protease. Emergency regulatory approval was obtained in Japan in November 2022. Post-marketing surveillance (PMS) is currently on-going to evaluate safety and effectiveness of ensitrelvir in Japanese real world clinical practice.

**Methods:**

In the PMS, 3000 COVID-19 patients who were administered ensitrelvir for the first time in about 500 Japanese hospitals or clinics are to be enrolled and observed for 28 days after the first administration. Patient demographics, concomitant medications, other therapy for COVID-19, adverse events, and clinical course (body temperature, presence or absence of systemic symptoms, respiratory symptoms, and gastrointestinal symptoms) are to be investigated.

**Results:**

Of the 234 cases collected by March 2023, 226 cases were included in the safety and effectiveness analysis population. Among the 226 cases, the average age was 43.5 years (the median age was 44.0 [12 - 91] years); 24 cases (10.6%) were aged 65 and over; and 115 cases (50.9%) were male. There were 44 cases (19.5%) with high-risk factors for severe illness. The severity of COVID-19 was mild in all cases.

Of the 226 cases in the safety analysis population, 34 events of non-serious adverse drug reactions were reported, and no cases/events of serious adverse drug reactions were reported. The major adverse drug reactions were diarrhea in 9 cases (4.0%), nausea in 4 cases (1.8%), and aggravation of bronchial asthma in 4 cases (1.8%). Two cases (0.9%) were hospitalized, and the reason for hospitalization was exacerbation of COVID-19 in both cases. No death cases were reported. The median time to resolution of fever was 36.0 hours in the standard-risk group (n=136) and 24.0 hours in the high-risk group (n=32). The median time to resolution of all symptoms was 144.0 hours in the standard-risk group (n=182) and 108.0 hours in the high-risk group (n=44).

In this presentation, results of the interim analysis on cases that have been collected by May 2023 will be reported.

**Conclusion:**

Results of the interim analysis suggest that ensitrelvir is well tolerated and effective in patients with or without risk factors.

**Disclosures:**

**Eriko Ogura, MD**, Shionogi & Co., Ltd.: I am an emplyee of Shionogi & Co., Ltd. **Noriko Nakagawa, n/a**, Shionogi & Co., Ltd.: I am an employee of Shionogi & Co., Ltd. **Noriko Hayashi, n/a**, Shionogi & Co., Ltd.: I am an emplyee of Shionogi & Co., Ltd.|Shionogi & Co., Ltd.: I am an emplyee of Shionogi & Co., Ltd. **Eri Tsukimura, n/a**, Shionogi Pharmacovigilance Center Co., Ltd.: I am an employee of Shionogi Pharmacovigilance Center Co., Ltd. **Satoru Takashima, n/a**, Shionogi & CO., LTD.: I'm an employee of Shionogi & CO., LTD.

